# Co-existence of Methanogenesis and Sulfate Reduction with Common Substrates in Sulfate-Rich Estuarine Sediments

**DOI:** 10.3389/fmicb.2017.00766

**Published:** 2017-05-05

**Authors:** Michal Sela-Adler, Zeev Ronen, Barak Herut, Gilad Antler, Hanni Vigderovich, Werner Eckert, Orit Sivan

**Affiliations:** ^1^Department of Geological and Environmental Sciences, Ben Gurion University of the NegevBeer-Sheva, Israel; ^2^Zuckerberg Institute for Water Research, The Jacob Blaustein Institutes for Desert Research, Ben Gurion University of the NegevBeer-Sheva, Israel; ^3^Israel Oceanographic and Limnological ResearchHaifa, Israel; ^4^Department of Earth Sciences, University of CambridgeCambridge, UK; ^5^The Yigal Allon Kinneret Limnological Laboratory, Israel Oceanographic and Limnological ResearchMigdal, Israel

**Keywords:** sulfate reduction, methanogenesis, substrates, estuaries, co-existence

## Abstract

The competition between sulfate reducing bacteria and methanogens over common substrates has been proposed as a critical control for methane production. In this study, we examined the co-existence of methanogenesis and sulfate reduction with shared substrates over a large range of sulfate concentrations and rates of sulfate reduction in estuarine systems, where these processes are the key terminal sink for organic carbon. Incubation experiments were carried out with sediment samples from the sulfate-methane transition zone of the Yarqon (Israel) estuary with different substrates and inhibitors along a sulfate concentrations gradient from 1 to 10 mM. The results show that methanogenesis and sulfate reduction can co-exist while the microbes share substrates over the tested range of sulfate concentrations and at sulfate reduction rates up to 680 μmol L^-1^ day^-1^. Rates of methanogenesis were two orders of magnitude lower than rates of sulfate reduction in incubations with acetate and lactate, suggesting a higher affinity of sulfate reducing bacteria for the available substrates. The co-existence of both processes was also confirmed by the isotopic signatures of δ^34^S in the residual sulfate and that of δ^13^C of methane and dissolved inorganic carbon. Copy numbers of *dsrA* and *mcrA* genes supported the dominance of sulfate reduction over methanogenesis, while showing also the ability of methanogens to grow under high sulfate concentration and in the presence of active sulfate reduction.

## Introduction

Estuarine and shallow shelf sediments are often characterized by high fluxes of nutrients, high loads of organic carbon and marine salinity, thus containing high sulfate concentrations and housing intensive bacterial sulfate reduction and methanogenesis. Estuarine sediments account for 10% of oceanic carbon emission rates, despite its relatively small area (Bange et al., 1994; Abril and Iversen, 2002). Nevertheless, our knowledge of the competition and co-existence between sulfate reduction and methanogenesis in these sediments is limited, and specifically it has yet to be determined whether they can co-exist while the active microbes sharing the same substrates. Furthermore, it is important to explore the conditions governing both the rates and initiation of methanogenesis in estuarine sediments. Here we examine the co-existence of methanogenesis and sulfate reduction while the microbes share the same substrates over a range of sulfate concentrations and rates of sulfate reduction through incubation experiments with sediment samples from the sulfate-methane transition zone of the Yarqon estuary (Israel).

The conventional paradigm states that thermodynamics govern biochemical depth profiles, and therefore in sedimentary environments, microbial processes that out-compete substrate uptake will suppress, or outcompete, other microbial processes, shifting the latter to greater depths in the sediment (Froelich et al., 1979; Stumm and Morgan, 1996). Despite this paradigm, a number of studies have shown that microbial processes can co-exist in complex sedimentary systems due to competition for electron donors rather than the difference in energy yield (Oremland and Taylor, 1978; Lovley et al., 1982). Examples of co-occurrence has been documented in the Black Sea sediments in which methane production was found within the sulfate reduction zone (Dale et al., 2008; Knab et al., 2008). Co-existence of sulfate reduction and methanogenesis characterizes also the coastal sediments of North Sea estuary. This co-existence was suggested to be controlled by the fast sediment accumulation combined with high organic carbon loading (Egger et al., 2016). These studies and others have emphasized that the various redox processes can co-exist in natural environments and may be coupled in a way that changes the rates of production or consumption of chemical species. These couplings would impact their distribution depth and their link to the subsurface carbon cycle. The co-existence between sulfate reduction and methanogenesis can occur at the interface between sulfate and methane, often termed the sulfate methane transition zone (SMTZ). Anaerobic oxidation of methane (AOM) coupled to sulfate reduction is typically found within this zone in marine sediments and has a large significance in controlling methane emission from marine sediment (Borowski et al., 2000; Archer, 2007; Knittel and Boetius, 2009). This process has been shown to consume up to 90% of the upward methane fluxes in marine sediments (Borowski et al., 1996; Valentine and Reeburgh, 2000).

Acetate and hydrogen are the preferred substrates for both sulfate reduction and methanogenesis (Schink, 1997; Conrad, 1999; Chidthaisong and Conrad, 2000a). From a thermodynamic perspective (Thauer et al., 1977; Schönheit et al., 1982; Ward and Winfrey, 1985; Lovley and Phillips, 1988) sulfate reducing bacteria can utilize hydrogen and acetate at lower concentrations than methanogens and therefore will likely outcompete them for substrate uptake, channeling the electron flow toward CO_2_ production rather than methane (Lovley et al., 1982; Oremland and Polcin, 1982; Lovley and Klug, 1983; King, 1984; Lovley and Goodwin, 1988). Sulfate reduction is known to restrict methanogenesis through several paths. In complex environments such as natural sediments, in the presence of sulfate reducing bacteria and methanogens, sulfate supplementation or high sulfate concentrations will inhibit methanogenesis, diverting the electron flow toward sulfate reduction (Mountfort et al., 1980; Mountfort and Asher, 1981).

Nevertheless, methanogenesis has been detected in zones dominated by sulfate reduction in marine and salt marsh sediments (Oremland and Taylor, 1978; Dale et al., 2008; Treude et al., 2014). This methanogenesis is assumed to be the product of non-competitive substrate uptake, (i.e., substrates that are consumed only by methanogens) such as methanol, methane thiol and methylamines (Oremland and Polcin, 1982; Kiene et al., 1986). Another mechanism that can explain the coexistence of methanogenesis and sulfate reduction is a cooperation between acetoclastic sulfate reducing bacteria that produce hydrogen and hydrogenotrophic methanogens (Ozuolmez et al., 2015). It can be also be a coupling between methanogens and fermentative (hydrogen producing) *Clostridia* (Oremland and Taylor, 1978) that may also support methane production in sulfate-enriched environments. On the other hand, inhibition of methanogenesis by sulfate reduction can be the result of the toxicity of sulfide, the product of sulfate reduction (Koster et al., 1986), even though one study suggested that the methanogen *Methanosarcina barkeri* could tolerate sulfide concentrations as high as 20 mM (Mountfort et al., 1980). Therefore, the conditions under which sulfate reduction and methanogenesis can co-exist in natural sedimentary environments and specifically in estuaries, and the possibility of these processes to share ambient substrates are still unclear. The goal of this study was to define the terms in which the methanogenesis and sulfate reduction co-exist using the highly stratified sulfate-enriched Yarqon estuary as a case study.

## Materials and Methods

### Study Site

**FIGURE 1:**
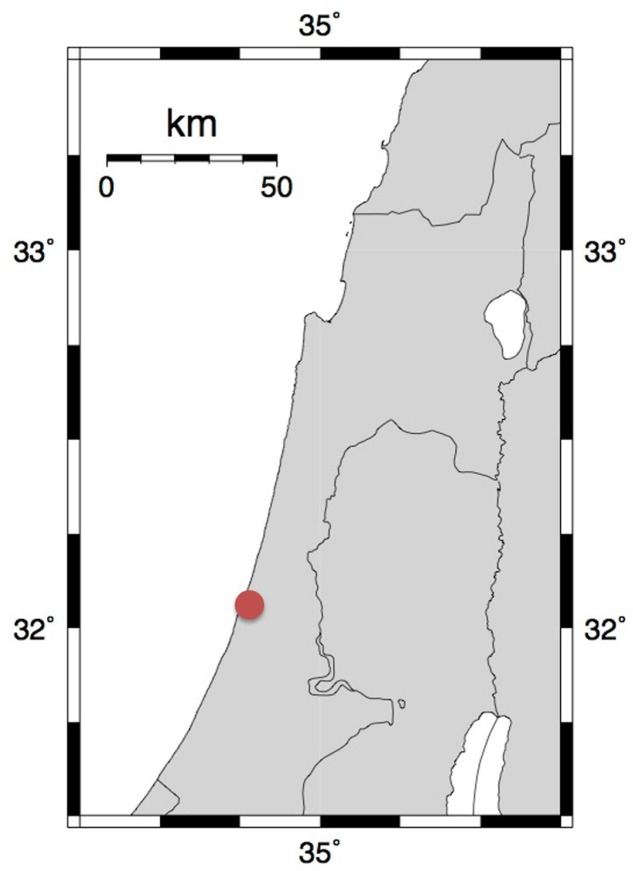
**Yarqon estuary location map at the Israeli coast of the Eastern Mediterranean**.

The Yarqon (**Figure 1**) is the largest coastal river in Israel with length of 27.5 km and a drainage basin area of 1800 km^2^. As other streams along the Mediterranean coast of Israel, the bottom bathymetry of the downstream lies below sea level, enabling the intrusion of seawater and the formation of highly stratified estuary up to a few kilometers inland. The estuary contains high organic carbon loads from upstream (20–60 mg L^-1^; Arnon et al., 2015) and lower water mass close to seawater salinity (∼19000 mg Cl^-^).

### Sediment Core Sampling

Sediment cores (∼35 cm long, 5 cm in diameter) were collected during August and October 2013 at the Yarqon estuary, 3 km upstream (32° 06.0792′ N; 34° 48.3633′ E), using a gravity corer as described in Antler et al. (2014). The cores were stored in the dark at 4°C and then sliced and treated within 48 h under anaerobic conditions.

### Experimental Design

Three incubation Experiments (A, B, and C- described below) were carried out using 1–3 replicates of sediments cores. Treatments parameters are outlined in **Table 1**. Each of the cores was sliced in the 5–15 cm depth interval under N_2_ flushing. Methane was measured from the head space using N_2_ pre-flushed gas tight syringe. Porewater sub-samples for sulfate and dissolved inorganic carbon (DIC) concentrations and isotopic measurements were extruded using N_2_ pre-flushed sterile 5 ml syringe (sub-sample of 2 ml).

**Table 1:** Description of Experiment A, B, and C with duplicate bottles for each treatment.

Experiment A	Sediment core collected during August 12th 2013; The goal of the experiment was to determine the effect of different sulfate concentrations on methane production rates, with and without sulfate reduction inhibitor			

Treatments	Sulfate concentration in slurry (mM)			

	100 μl of ^13^CH_4_ (99.999%) was added to all slurries			

No inhibitor	9 mM	2 mM	1 mM	Killed control + 9 mM
Sulfate reduction inhibitor	9 mM + Molybdate	2 mM + Molybdate	1 mM + Molybdate

**Experiment B**	Sediment core collected during October 1st 2013; The goal of the experiment was to determine the effect of inhibitors addition on methane production and effect of sulfate reduction rates and isotopic signature			

	10 mM sulfate without substrate addition in all slurries Inhibition conditions			

Treatments	No inhibitor addition-control	20 mM BES	10 mM Molybdate

**Experiment C**	Sediment core collected during October 1st 2013; The goal of the experiment was to determine the effect of substrate and the effect of inhibitors addition on methane production and sulfate reduction rates			

Treatment Substrate conditions	10 mM sulfate in all slurries Inhibition conditions			

40 mM acetate	No inhibitor	20 mM BES	Molybdate	Killed control with acetate addition
10 mM lactate	No inhibitor			


*Experiment A*-(**Table 1**) was conducted on a sediment core from August 2013. The sub-sampled sediment was homogenized and approximately 30 gr of sediment was transferred to N_2_ pre-flushed 300 ml sterile glass bottles. Sterile and anaerobic 3.5% NaCl omit solutions were pre-prepared with different sulfate concentrations (1, 2, or 9 mM), with or without molybdate (MoO_4_^2-^) as a sulfate reduction inhibitor (Oremland and Capone, 1988). The range of sulfate concentration and the layer isolated were chosen based on *in situ* sulfate and methane profiles that show that sulfate reduction and methanogenesis overlap in the Yarqon with sulfate concentration up to 10 mM (Antler et al., 2014). The sediment was mixed with the media at a 1:4 ratio to produce slurry and closed with black butyl rubber stoppers. Three times in sequence, bottles were shaken vigorously for 30 s followed by flushing with a N_2_ + 300 ppm CO_2_ mixture for 5 min at the beginning of the experiment. Labeled ^13^C methane was added to all slurries at a concentration of 100 μmol L_slurry_^-1^. For each treatment duplicates were prepared. Killed control bottles were autoclaved after the bottles were sealed.

*Experiment B and C*-(**Table 1**) were conducted on three sediment cores retrieved on October 2013 that were sliced in the depth interval of 10–25 cm depth in an anaerobic hood (Coy Lab-Grass Lake, MI, USA). The sediment slices were homogenized and mixed with sterile and anaerobic medium solutions that were prepared in advance with 3.5% NaCl and 10 mM sulfate, in a 1:4 sediment: medium ratio. One hundred and twenty mililiter sub samples from each slurry were transferred into 300 ml sterile glass bottles and closed with black butyl rubber stoppers inside the anaerobic hood. Three times in sequence, bottles were shaken vigorously for 30 s followed by flushing with a N_2_ + 300 ppm CO_2_ mixture for 5 min at the beginning of the experiment. *Experiment B* was conducted on slurries treated with 10 mM molybdate as a sulfate reduction inhibitor or with 20 mM 2-bromoethanosulfonate (BES; Sigma–Aldrich, Rehovot, Israel) as a methanogenesis inhibitor (Chidthaisong and Conrad, 2000b) or without an inhibitor (as a control). All slurries in Experiment B were not amended with a substrate. *Experiment C* was conducted on slurries treated with substrate and inhibitors addition. The substrate additions were 7 mM acetate or 7 mM lactate. Killed control bottles were autoclaved after sealing and substrate was added. For each substrate, two bottles were treated with 20 mM of BES or 10 mM molybdate or with no inhibitor addition. Duplicate bottles were made for each treatment.

### Analytical Methods

#### Chemical Analyses

Headspace methane concentrations were measured on a gas chromatograph equipped with a flammable ionization detector (FID) at a precision of 2 μmol CH_4_ L^-1^. Sulfate was measured after porewater were filtered with a 0.22 μm filter and diluted by a factor of ∼1:100 (by weight using a Dionex DX500 high pressure liquid chromatograph (HPLC) with an error of 3%. δ^13^C_DIC_ was measured in ∼0.5 mL of each sample. The sample was transferred into a He-flushed vial containing 50 μl of concentrated H_3_PO_4_ that released all DIC to the headspace as CO_2_. Measurements of the released CO_2_ was done using a conventional isotopic ratio mass spectrometer (IRMS, DeltaV Advantage, Thermo) with a precision of ±0.1‰, and the results are reported versus the Vienna Pee Dee Belemnite (VPDB) standard. DIC concentration was calculated from the IRMS results according to peak height and to a calibration curve (by standard samples prepared from NaHCO_3_) with an error of ±0.2 mM. The δ^13^C_CH4_ values were measured using an IRMS equipped with a PreCon interface after oxidation to CO_2_. The error between duplicates of this parameter was less than 0.5‰ and the results are reported versus the VPDB standard. For δ^34^S_SO4_ analysis, sulfate was precipitated as barium sulfate (barite) using a saturated barium chloride solution (as described in Antler et al., 2014). The barite was then washed with 6N HCl and distilled water. The barite was combusted at 1030°C in a Flash Element Analyzer (EA), and the resulting sulfur dioxide (SO_2_) was measured by continuous flow on a GS-IRMS (Thermo Finnegan Delta V Plus Godwin Laboratory, University of Cambridge). The error for δ^34^S_SO4_ was determined using the standard deviation of the standard NBS 127 at the beginning and the end of each run (∼0.3‰ 1σ). Samples were corrected to NBS 127, IAEA-SO-5 and IAEA-SO-6 standards (20.3, 0.5, and -34.1‰, respectively). The δ^34^S_SO4_ values are reported versus Vienna Canyon Diablo Troilite (VCDT). Data analysis of variance (single factor ANOVA) test was conducted on concentration measurements of methane and sulfate to test the variance between the treatments described above with α = 0.05.

#### Quantitative Polymerase Chain Reaction (qPCR) Analyses

Reaction mix for qPCR included the following: 12.5 μl KAPA SYBR Fast Universal Ready mix (KAPA Biosystems, Woburn, MA, USA); 100 nM of each primer, 1 μl template (extracted DNA or plasmid) and DDW to complete to 25 μl. Thermocycling conditions included an initial denaturation step at 95°C, followed by 40 cycles of 95°C for 30 s; annealing temperatures as described in **Table 2** for 30 s; and 72°C for 30 s. Acquisition was performed at the completion of each cycle, following a short (2 s) step at 78°C to ensure primer dimer denaturation. Melting curves (72–95°C) showed only one peak for all qPCR reactions. PCR for *mcrA* (methanogens), *dsrA* (sulfate reducing bacteria), and 16S rRNA genes of archaea and bacteria were preformed based on Wilms et al. (2006) and Yu et al. (2008). Calibration curves for mcrA, dsrA, archaea and bacteria were created by conducting a 10-fold dilution series (∼10^3^–10^9^ copies) of plasmids (constructed as detailed below), containing environmental copies of the relevant genes. For calibration of 16s rRNA genes, genomic DNA from a pure culture of *Escherichia coli* was used, assuming that the 16S copy number in this genome is 6. Calibration curves had R^2^ > 0.975, and the slope was between -3.0 and -3.9, corresponding to PCR efficacy of 90–111%. The copy number of *mcrA* was normalized to archaea copy number and the copy number of *dsrA* was normalized to bacteria copy number in the same sample. Amplification reactions were carried out in a Rotor-Gene^TM^ 6000 thermocycler (Corbett Life Science, Concorde, NSW, Australia). Primer sequences are detailed in **Table 2**.

**Table 2:** Primers and annealing temperature based on ^∗^Wilms et al., 2006 and ^∗∗^Yu et al., 2008.

Primer name		Annealing temp	Length (bp)
dsrA^∗^	5′-ACSCACTGGAAGCACG-3′	58°C	450
	5′-CGGTGMAGYTCRTCCTG-3′		
mcrA^∗^	5′-GCMATGCARATHGGWATGTC-3′	54°C	350
	5′-TGTGTGAASCCKACDCCACC-3′		
Archaea^∗∗^	5′-ACGGGGYGCAGCAGGCGCGA-3′	48°C	320
	3′-GTGCTCCCCCGCCAATTCCT-5′		
Bacteria^∗^	5′-CCTACGGGAGGCAGCAG-3′	60°C	270
	3′-ATTACCGCGGCTGCTGG-5′		


## Results

### Experiment A

**FIGURE 2:**
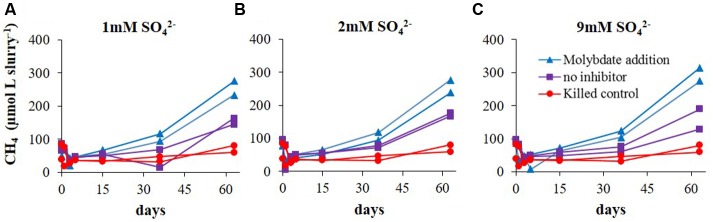
**Experiment A – Evolution of methane concentrations in slurries with** **(A)** 1 mM ${SO}_{4}^{2-}$ ; **(B)** 2 mM ${SO}_{4}^{2-}$ ; and **(C)** 9 mM ${SO}_{4}^{2-}$.

**FIGURE 3:**
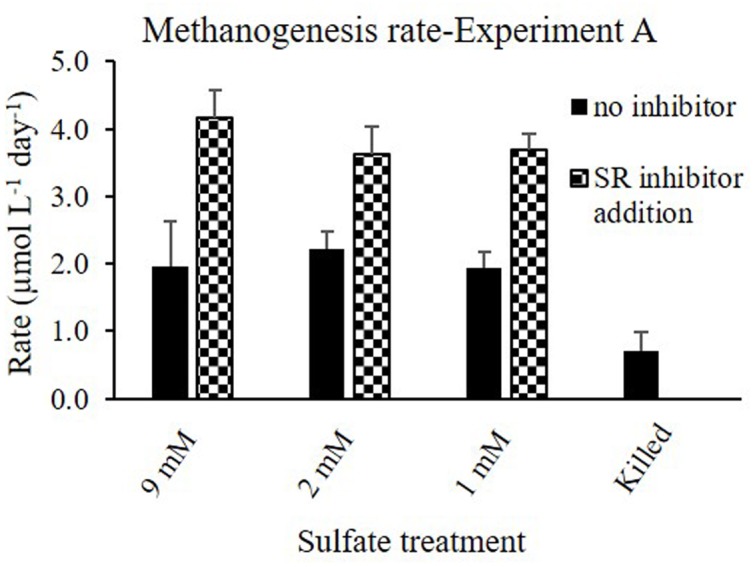
**Methanogenesis rates in Experiment A with and without sulfate reduction inhibitor (molybdate) under different sulfate concentrations**.

The first set of experiments (Experiment A) aimed to examine the effect of sulfate concentration on the rate of methanogenesis and the lag time for its initiation. Both, methanogenesis rates and the lag time of methanogenesis initiation were similar (40 days) regardless of sulfate concentrations (9, 2, and 1 mM sulfate; **Figure 2**). In this experiment the effect of sulfate reduction on methanogenesis was tested as well. This was done by molybdate addition, a sulfate reduction inhibitor. In all the non-inhibited slurries, methane concentrations reached ∼170 μmol L_slurry_^-1^, and even slightly higher values with 9 mM sulfate. As expected, when molybdate was added to the slurries, methanogenesis was stimulated and methane concentrations increased up to 250–300 μmol L_slurry_^-1^ in all slurries. In the killed control (with 9 mM sulfate) methane concentrations did not increase throughout the experiment (**Figure 2**). A comparison between methanogenesis rates in the range of sulfate concentrations with and without sulfate reduction inhibitor is presented in **Figure 3**.

**FIGURE 4:**
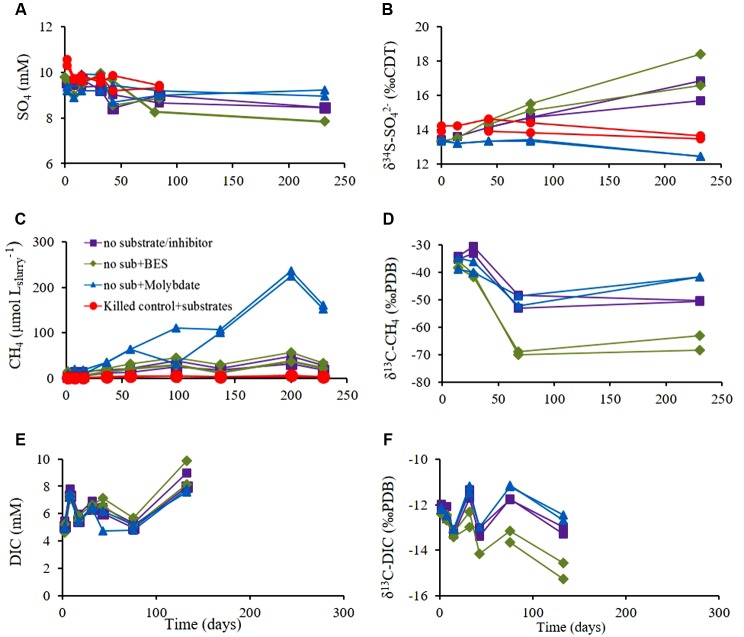
**Experiment B – Slurries with sediment amended with inhibitors of either sulfate reduction (molybdate) or methanogenesis (BES), control slurries (without inhibitor) and killed control (autoclaved).** **(A)** Sulfate concentration; **(B)** δ^34^S of residual ${SO}_{4}^{2-}$ ; **(C)** CH_4_ concentrations; **(D)** δ^13^C-CH_4_; **(E)** DIC concentrations and **(F)** δ^13^C-DIC. Legend in panel **(A)** refers to all panels.

During this experiment sulfate concentrations decreased by 0.1 – 0.3 mM, indicating low reduction rates. DIC concentrations increased by ∼0.9 mM when molybdate was added and by ∼1.0 mM in non-inhibited slurries. This difference is not significant as it is in the range of the standard error. The difference in the δ^13^C_DIC_ values between the treatments was also not significant, and remained between -16‰ to -18‰. Although ^13^C labeled methane was added to all slurries (initial concentration of 100 μmol L_slurry_^-1^), ^13^C enriched DIC was not detected in slurries, indicating that AOM was insignificant in this short time scale experiments, as was shown in marine and lake sediments (Sivan et al., 2014; Bar-Or et al., 2015). Data of this experiment was not shown as it was similar to Experiment B, which was fuller and presented in **Figure 4**.

### Experiment B

The effect of sulfate reduction on methanogenesis was examined by treatment with molybdate; and the effect of methanogenesis on sulfate reduction was examined by treatment with BES (a methanogen inhibitor) in slurries. The results of Experiment B without inhibitors supplementation (**Figure 4**) were similar to those of Experiment A. Sulfate concentrations in the non-inhibited and BES-treated slurries decreased by a factor of 5–10 relative to molybdate-treated slurries, demonstrating the efficiency of molybdate as an inhibitor of sulfate reduction (**Figure 4A**). The values of δ^34^S_SO4_ increased by 2.3–5.1‰ in non-inhibited slurries and BES-treated slurries, and decreased in slurries treated with molybdate and in the killed controls by 0.4–0.9‰, (**Figure 4B**).

Methane concentrations increased in all treatments. As expected, the maximum increase was observed in slurries treated with molybdate and the minimum increase was observed in slurries treated with BES and in the non-inhibited slurries (**Figure 4C**). The initial value of δ^13^C_CH4_ in all slurries was approximately -35‰, and decreased throughout the experiment. Maximum depletion was observed in BES-treated slurries (methane production in BES-treated slurries and non-inhibited slurries showed similar rates) and minimum depletion was observed in slurries treated with molybdate, corresponding to methanogenesis rates in the slurries (**Figure 4D**).

The DIC concentrations were similar in all slurries and increased only by approximately 3 mM (**Figure 4E**). The initial DIC concentrations were 4.6–5.4 mM in all slurries and the initial δ^13^C_DIC_ value in all slurries was ∼-12‰. This value slightly decreased during the experiment with a similar trend as δ^13^C_CH4_ with maximum depletion in the BES-treated slurries, and minimum depletion in molybdate-treated slurries (**Figure 4F**).

### Experiment C

**FIGURE 5:**
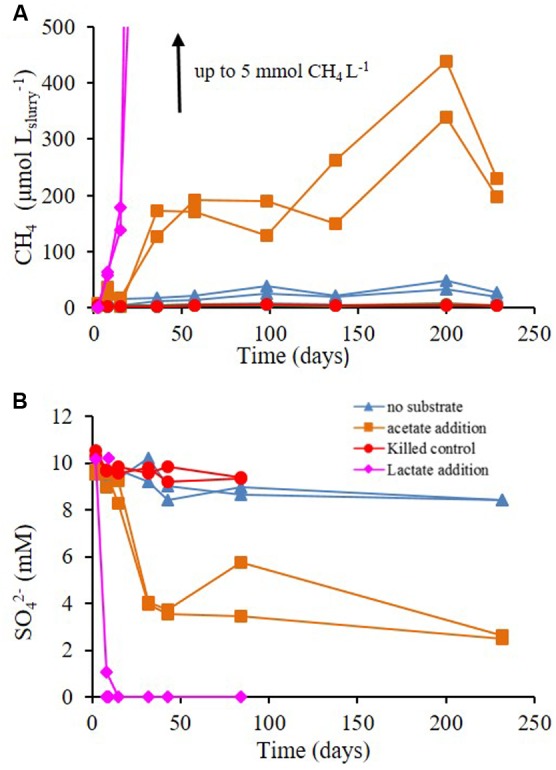
**Experiment C –** **(A)** methane and **(B)** sulfate concentrations throughout the experiment with no substrate addition, acetate addition, lactate addition and killed control (autoclaved).

**FIGURE 6:**
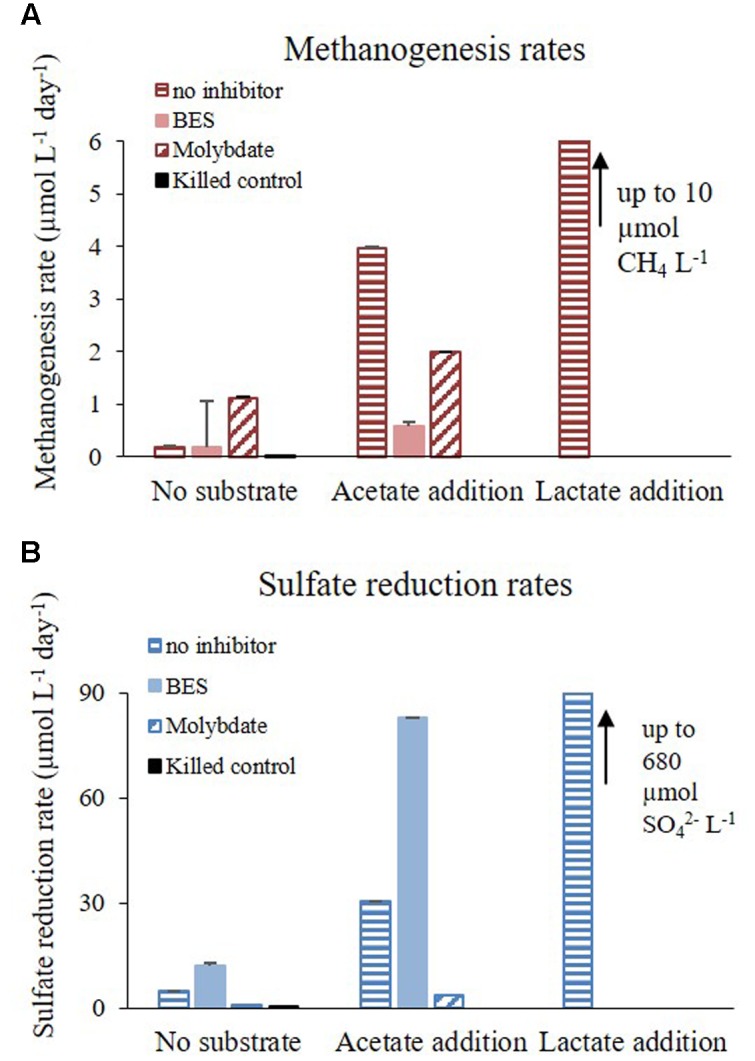
**Rates of** **(A)** methanogenesis and **(B)** sulfate reduction during Experiments B and C treated with inhibitors (BES, molybdate or without inhibitor) and with substrate (acetate, lactate or without substrate addition).

The effect of acetate and lactate supplements on methane production and sulfate reduction rates was examined during Experiment C. These additions stimulated methane production in all slurries. The maximum increase in methane concentration was observed in slurries treated with lactate and in decreasing order with acetate addition, no substrate supplementation and finally killed control (**Figure 5A**). The most prominent decrease in sulfate during the experiment was in the slurries supplemented with lactate, where sulfate completely depleted within 5 days; followed by slurries supplemented with acetate, and those without substrate supplementation (**Figure 5B**). The effect of inhibitors and substrates addition on sulfate reduction and methanogenesis rates in Experiments B and C is shown in **Figure 6**.

**FIGURE 7:**
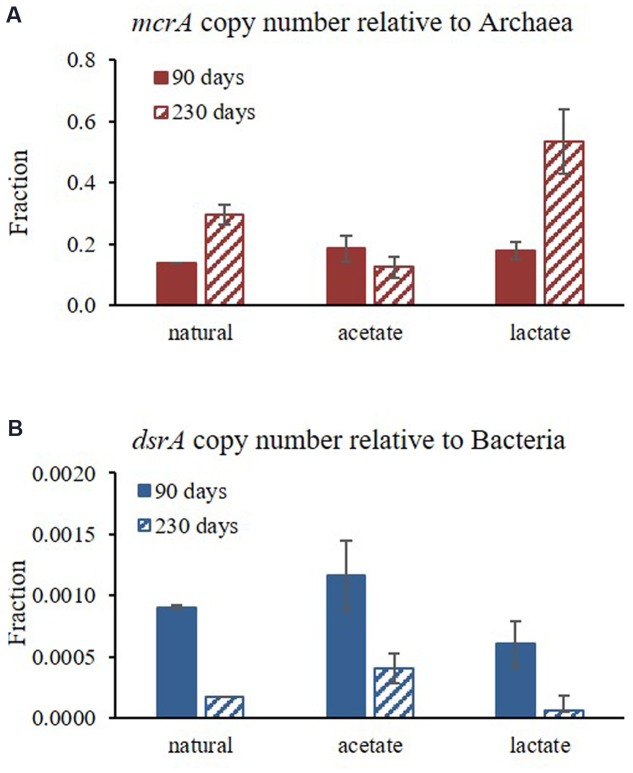
**Changes in the relative abundance of** **(A)** *mcrA* functional gene relative to the copy number of Archaea; and **(B)** *dsrA* functional gene relative to the copy number of Bacteria over the course of the experiment at two time points (average of duplicate bottles (each samples in triplicates).

The qPCR results for the abundances of *mcrA* (normalized to copy number of archaea) and *dsrA* (normalized to the copy number of bacteria) functional genes at two time points in Experiment C (after 90 and 230 days) are presented in **Figure 7**. The results show that methanogens (based on *mcrA* gene) under natural conditions constituted approximately 10% of all archaea, and sulfate reducing bacteria (based on *dsrA* gene) were approximately 0.1% of all bacteria (**Figure 7** and **Table 3**). In general, sulfate reducing bacteria were more abundant during the first step of the experiment and methanogens became more abundant toward the end of the experiment (**Figure 7**).

**Table 3:** Copies per gram dry sediment of specific genes for each of the duplicate bottles.

	Copies per gr dry sediment									

Natural sediment at t_0_	Archaea		Bacteria		Methanogens		Sulfate reducing bacteria		Archaea/Bacteria ratio	
	2.4 × 10^8^		1.0 × 10^11^		3.4 × 10^7^		7.6 × 10^7^		2.4 × 10^-3^	
					
Treatment	90 days	230 days	90 days	230 days	90 days	230 days	90 days	230 days	90 days	230 days
Natural	7.3 × 10^8^	1.7 × 10^8^	7.8 × 10^11^	4.7 × 10^11^	1.0 × 10^8^	4.4 × 10^7^	7.0 × 10^8^	8.3 × 10^7^	9.3 × 10^-4^	3.6 × 10^-4^
	6.0 × 10^8^	1.5 × 10^8^	4.9 × 10^11^	4.2 × 10^11^	8.3 × 10^7^	4.9 × 10^7^	4.5 × 10^8^	7.2 × 10^7^	1.2 × 10^-3^	3.6 × 10^-4^
Acetate	9.3 × 10^8^	3.7 × 10^8^	2.6 × 10^12^	1.5 × 10^12^	2.1 × 10^8^	5.8 × 10^7^	2.3 × 10^9^	7.9 × 10^8^	3.6 × 10^-4^	2.5 × 10^-4^
	4.8 × 10^8^	1.8 × 10^8^	6.8 × 10^11^	8.6 × 10^11^	6.8 × 10^7^	1.6 × 10^7^	9.9 × 10^8^	2.4 × 10^8^	7.1 × 10^-4^	2.0 × 10^-4^
Lactate	4.6 × 10^9^	1.9 × 10^9^	4.8 × 10^12^	2.7 × 10^12^	9.4 × 10^8^	8.3 × 10^8^	3.8 × 10^9^	1.5 × 10^8^	9.6 × 10^-4^	7.2 × 10^-4^
	8.1 × 10^9^	2.7 × 10^9^	7.0 × 10^12^	4.7 × 10^12^	1.2 × 10^9^	1.8 × 10^9^	2.9 × 10^9^	3.1 × 10^8^	1.2 × 10^-3^	5.8 × 10^-4^


## Discussion

Sulfate penetration into the methane production zone was already observed in diverse environments. For example, in continental margin sediments (Treude et al., 2014), in the gassy sediments of Eckernforde Bay in the German Baltic (Treude et al., 2005), in littoral Baltic Sea sediments (Thang et al., 2013), in the saline coastal sediments of Lake Grevelingen in the Netherlands (Egger et al., 2016), in the sediments of the Black Sea (Knab et al., 2009) and in salt marsh sediments (Parkes et al., 2012). However, couplings between sulfate concentrations, sulfate reduction rates and methanogenesis rates have not been fully examined in estuarine sediments. Previous porewater data from sediments of the Yarqon estuary documented a combination of a high organic matter loading and seawater sulfate concentrations, triggering relatively high rates of sulfate reduction (6.05 × 10^-4^ mol L^-1^ day^-1^) with maximum values at the upper 5 cm sediment depth. These were relatively high rates compared to other marine or saline estuarine environments (Eliani-Russak et al., 2013). Methane accumulated to above 300 μmol L^-1^ in the sulfate reduction zone when sulfate concentrations were above 3 mM at sediment depth of ∼10 cm. In addition, sulfate was not completely exhausted until 20 cm below surface, penetrating into the methanogenesis zone. The isotopic signature of oxygen (δ^18^O_SO4_) versus sulfur (δ^34^S_SO4_) in dissolved sulfate profiles indicated that in the Yarqon estuary sediment (during May 2010), AOM is likely the main sulfate reduction process (Antler et al., 2014). In this study we used sediment cores from the saline Yarqon estuary at the zone identified as containing both processes.

### Sulfate Concentration Effect

The experiments in this research were conducted under a sulfate concentration range of 1–10 mM. This specific concentration range was chosen for several reasons: (1) *In situ* evidences show that sulfate reduction and methanogenesis overlap in the Yarqon and that sulfate concentrations in this depth can reach 10 mM (Antler et al., 2014); (2) Different sulfate and substrate concentrations cause different reduction rates in the sediment; (3) Flood events cause the salinity gradient to retreat toward seashore, which changes the sulfate concentration gradient.

Methanogens activity is not affected by sulfate concentrations, as was shown in pure cultures (King, 1984). Nevertheless, in different sedimentary systems, the competition with sulfate reducing bacteria for labile substrate controls methanogenesis, since sulfate reducing bacteria outcompete methanogens for substrate uptake. Elevated sulfate concentration will cause an enhancement in sulfate reduction rates and therefore a decrease in methanogenesis, and vise versa – low sulfate concentrations will decrease sulfate reduction rates and will enhance methanogenesis (Mountfort et al., 1980; Lovley et al., 1982; Lovley and Klug, 1983). Here we show that even at elevated sulfate concentrations in sedimentary systems, sulfate reduction and methanogenesis co-exist, and the rates of sulfate reduction control methanogenesis rates. The negligible effect of sulfate concentration on the methanogenesis rates in slurries indicates that the concentration itself is not the direct controlling factor of methanogenesis. The amendments of the slurries with sulfate at various concentrations (Experiment A; **Figure 2**) did not influence sulfate reduction rates either, indicating that this system is probably substrate depleted. Furthermore, supplementation of molybdate, which lowered the rates of sulfate reduction, enhanced methane production in all slurries (**Figure 3**). Therefore, we suggest that the competition between methanogens and sulfate reducing bacteria for a common substrate is the main factor controlling the rates and onset of methanogenesis.

### Isotopic Effect

Microorganisms tend to discriminate against the heavy isotope, leaving the product isotopically enriched in the light isotope and the residual pool in the heavy isotope. Sulfate reduction and methanogenesis processes have large isotope fractionation effect, and thus related isotope measurements may be more distinct and sensitive than the measurement of concentration changes (e.g., Sivan et al., 2014). Dissimilatory sulfate reduction is characterized by large isotopic fractionation (up to 72‰) with the light isotope favored in the H_2_S product (Kaplan and Rittenberg, 1964; Canfield, 2001; Wortmann et al., 2001; Sim et al., 2011). In experiment B there was a significant increase in δ^34^S_SO4_ in the slurries without inhibitor and in the slurries with the BES addition. A small decrease was observed in the killed control and in the experiment with molybdate addition (**Figure 4B**). This indicates active sulfate reduction in the slurries without inhibitor and with the BES addition (the change in sulfate concentration is small during the course of the experiment as it is less sensitive). The decrease in δ^34^S_SO4_ in the killed control and in the experiment with molybdate supplementation toward the end of experiment can be attributed to anaerobic abiotic oxidation of reduced sulfur compounds (Balci et al., 2007). Furthermore, sulfate reduction was enhanced when methanogenesis was inhibited (by BES) (**Figure 6B**) and methanogenesis rate was enhanced when sulfate reduction was inhibited (by molybdate) (**Figure 6A**). It seems therefore that the enrichment of ^34^S of residual sulfate is correlated with the rate of sulfate reduction, as shown previously (Kaplan and Rittenberg, 1964; Habicht and Canfield, 1997; Sim et al., 2011).

During methanogenesis the carbon fractionation against ^13^C is about 25–90‰, producing very light methane with δ^13^C_CH4_ of -50 to -100‰ and enriched δ^13^C_DIC_ (e.g., Ferry, 1992; Whiticar, 1999; Gelwicks et al., 1994). The fractionation varies among the different pathways of methanogenesis. In hydrogenotrophic methanogenesis, CO_2_ is reduced by H_2_, the fractionation is slightly larger than the fractionation of acetoclastic methanogenesis and can exceed 55‰. In acetoclastic methanogenesis methane is derived from the methyl group of acetate, the fractionation is slightly lower and ranges between 40 and 60‰ (Whiticar et al., 1986; Whiticar, 1999).

Slurries treated with BES and non-inhibited slurries showed similar methanogenesis rates, based on methane concentrations measurements. However, the isotope measurements, which are often more sensitive, showed significant stronger depletion of δ^13^C_CH4_ in slurries treated with BES relative to non-inhibited slurries (**Figures 4D,F**). Valentine et al. (2004) and Penning et al. (2005) showed that during hydrogenotrophic methanogenesis high levels of H_2_ were correlated with low fractionation in carbon, and low levels of H_2_ were correlated with higher fractionation. The authors hypothesized that this difference is controlled by the extent of the enzymatic reversibility which, is controlled by the Gibbs free energy of catabolism, similar to dissimilatory sulfate reduction (Kaplan and Rittenberg, 1964; Habicht and Canfield, 1997). BES is an analog of coenzyme M found in all methanogens and is a specific inhibitor for methanogens (Chidthaisong and Conrad, 2000b). Since coenzyme M is involved in the rate limiting step in methane production (Scheller et al., 2013), we propose that BES addition stops the reversibility of methanogenesis or acts similarly to low levels of H_2_ or reduced Gibbs free energy (less negative) and therefore the fractionation is larger in the BES treated slurries.

### Substrate Supplementation Effect

**FIGURE 8:**
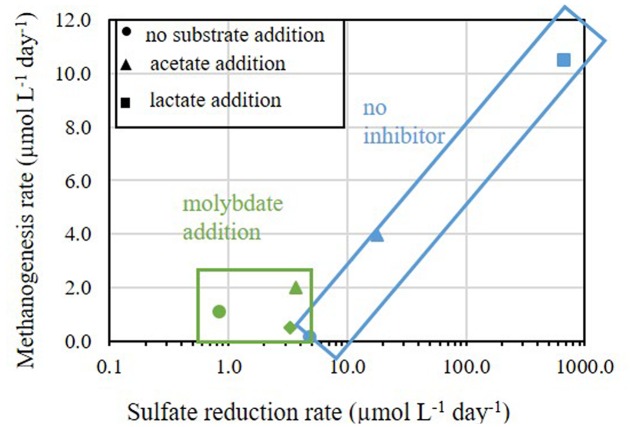
**Methanogenesis rates versus sulfate reduction rates in Experiments B and C.** The rates of samples without addition of inhibitors are marked in blue symbols and rectangle and the rates of samples treated with molybdate are marked in green symbols and rectangle.

Methanogenesis and sulfate reduction rates calculated from Experiment C show that the supplementation of lactate and acetate had a significant effect on the rate of the processes (**Figure 6**). Similarly to Experiment A, when a sulfate reduction inhibitor (molybdate) was added methanogenesis was enhanced, with the exception of acetate supplementation (**Figure 6A**). The rates of sulfate reduction and methanogenesis increased by one order of magnitude as a result of acetate supplementation, and with lactate both rates increased by two orders of magnitude (**Figure 8**). Although the effect of a non-competitive substrate was not examined in this research, the results show that competitive labile organic matter had similar effects on the rates of sulfate reduction and methanogenesis. This indicates that at a high organic load, sulfate reduction and methanogenesis can co-exist and share ambient electron donors (Ozuolmez et al., 2015; Egger et al., 2016). The effect of labile organic matter on methanogenesis rate was shown also in a recent study from a shallow sediment in the Peruvian margins having co-existence of methanogenesis and sulfate reduction with 1–2 order of magnitudes difference in rates, which remain steady along the organic carbon concentration gradient (Maltby et al., 2015).

Nevertheless, when considering the absolute rates, sulfate reduction was the favorable process over methanogenesis in slurries with substrate supplementation. This advantage could be attributed to thermodynamic preference of sulfate reduction compared to methanogenesis (Lovley and Goodwin, 1988) and the population composition under natural conditions, in which sulfate reducing bacteria represented 10% of the total microbial community while methanogens represented less than 0.01% (**Table 3**). This is shown in **Figure 8**, which summarizes the effect of substrate addition on the rates of sulfate reduction and methanogenesis. When lactate and acetate were added both rates were enhanced by two and one orders of magnitude, respectively. Nevertheless, a two orders of magnitude difference between sulfate reduction and methanogenesis remained. Thus, it appears that the competition over substrate controls the intensity of methanogenesis in estuarine sediments (**Figure 8**).

According to the microbial population analysis, sulfate reducing bacteria were dominant at the beginning of Experiment B, whereas over the course of the experiment methanogen abundance increased, even though sulfate concentrations was still high with acetate supplementation (7 mM; **Figure 7**). In addition, lactate addition stimulated rapid sulfate reduction, which may have caused sulfate reducers to be sulfate-limited and enabled methanogens to strengthen. This suggests that sulfate reducing bacteria have an initial advantage for electron donor uptake; however, methanogens can also grow under elevated sulfate concentration and sulfate reduction rates. This observation strengthens our hypothesis that sulfate reduction and methanogenesis can co-exist and initial sulfate concentrations in the sediment do not control methane production initiation and intensity. The rate and initiation depth of methanogenesis is probably more strongly affected by the competition and the quantitative advantage of the sulfate reducing bacteria over the methanogens, as well as their stronger affinity for substrate uptake and the fact that sulfate reducing bacteria are not sulfate limited in these sediments (**Figure 7**).

Sulfate reducing bacteria have higher affinity to hydrogen and acetate than methanogens (Oremland and Polcin, 1982). However, co-existence of sulfate reduction and methanogenesis was shown under substrate limited conditions and following substrate supplementation, without dependence on sulfate concentration in the Yarqon sediments. The qPCR results show that acetate supplementation considerably enhanced the abundance of sulfate reducing bacteria and may indicate that the dominant electron donor in this process in the sediments is acetate. Lactate supplementation enhanced methanogens considerably, probably due to hydrogen production during lactate degradation and due to quick depletion in sulfate concentrations in the slurries treated with lactate.

## Summary

This study evaluated the regulatory effects of sulfate concentrations and microbial sulfate reduction on methanogenesis in the SMTZ of estuarine sediments using the Yarqon river estuary as a case study. The results show that: (a) Sulfate concentrations do not limit the onset and methanogenesis rates in the Yarqon estuarine sediments, even when sulfate concentrations are as high as 10 mM; (b) The main factors controlling methanogenesis initiation and intensity are sulfate reduction rate and substrate availability and; (c) Methanogenesis can co-exist with sulfate reduction in a large range of sulfate reduction rates (5–700 μmol L^-1^ day^-1^) that are controlled by substrate and inhibitor additions. Sulfate reducing bacteria have a distinct favorable substrate utilization, as apparent by two orders of magnitude higher reduction rates compared to methanogenesis rates. The qPCR analysis results strengthen our geochemical data and show a shift in time from an initial dominance of sulfate reducing bacteria to a growth of the methanogen community toward the end of the experiment. Although estuarine sediments represent only 0.7% of the total marine sediments area, they contribute 7–10% of oceanic emissions of carbon to the atmosphere (Bange et al., 1994; Abril and Iversen, 2002). Thus, studying methane production controls and specifically the co-existence of the two main carbon sink processes in estuarine sediments is globally important. The results from the Yarqon estuary may be significant to other estuarine environments that show co-existence of methanogenesis and sulfate reduction.

## Author Contributions

MS-A and OS designed the experiments, MS-A and HV performed the work, GA measured the sulfur isotopes. MS-A, ZR, BH, GA, WE, and OS analyzed the data and wrote the manuscript.

## Conflict of Interest Statement

The authors declare that the research was conducted in the absence of any commercial or financial relationships that could be construed as a potential conflict of interest.
